# Healthcare costs in patients with head and neck cancer

**DOI:** 10.1007/s00520-026-10608-y

**Published:** 2026-03-31

**Authors:** Justin Smith, Madhavi Chilkuri, Daniel Lindsay, Katharina M. D. Merollini

**Affiliations:** 1 Townsville University Hospital, Townsville, QLD Australia; 2 James Cook University College of Medicine and Dentistry, Townsville, QLD Australia; 3 QIMR Berghofer Medical Research Institute, Brisbane, QLD Australia; 4 University of Queensland, Brisbane, QLD Australia; 5 School of Health, University of the Sunshine Coast, Sippy Downs, QLD Australia; 6 Sunshine Coast Health Institute, Sunshine Coast University Hospital, Birtinya, QLD Australia

**Keywords:** Head and neck cancer, Healthcare costs, Cost of care, Medical costs, Australia

## Abstract

**Purpose:**

The aim of this study was to quantify the healthcare costs of patients with head and neck cancer (HNC) in Australia and investigate factors associated with increased costs.

**Methods:**

A sub-study was performed using linked patient data from ‘Lifetime Costs of Surviving Cancer – A Queensland Study (COS-Q).’ Queensland residents diagnosed with a first primary mucosal head and neck squamous cell carcinoma (HNSCC) between 1997 and 2015 and who incurred costs from 2013 to 2016 were included. Healthcare costs were reported as a total mean cost per patient per year and included emergency department (ED) costs, hospital admission costs, cost for medical and allied health services and pharmaceutical costs.

**Results:**

There were 4929 patients with HNSCC included in the study. The total mean annual cost was $21,646 per patient with the highest costs in the first-year post treatment. Regional ($23,312) and rural ($33,627) patients experienced higher healthcare costs than those from major cities ($20,300, p < 0.001). Factors strongly associated with an increased overall healthcare cost included living in a remote location (RR 1.51, p < 0.001), having a nasopharynx (RR 1.41, p = 0.027) or hypopharynx (RR 1.60, p < 0.001) cancer or never married (RR 1.34, p < 0.001). There were 644 patients (13%) who developed a second primary malignancy and this sub-group experienced higher overall costs ($46,453 vs $24,966, p < 0.001).

**Conclusion:**

Healthcare costs for patients with HNC are significant. Further research is needed to investigate ways to reduce healthcare utilisation and provide additional support in this cohort.

## Introduction

Head and neck cancer (HNC) is a diverse group of tumours that requires significant multi-disciplinary input [1]. Management of HNC has been shown to incur significant societal costs, including direct medical care costs and indirect costs such as lost productivity and premature mortality [2]. The medical costs of care include not only the initial management of the index HNC but also costs during the follow-up period where these patients may experience a HNC recurrence, second primary tumour or ongoing side effects from their treatment.

Previous studies have demonstrated varying medical costs for patients with HNC, depending on methodology utilised and the healthcare setting in which they were conducted. Most studies have been performed in the USA with wide variations in reported costs [3–5]. A European study of 879 HNC patients found that direct health care costs in the first 2 years of diagnosis were €20,184 [6]. Only one study has previously reported the healthcare costs of HNC in Australia, and this was a retrospective study of 113 patients with oral squamous cell carcinoma (SCC) [7].

Quantifying costs for patients with HNC will allow patterns of healthcare utilisation in Australia to be explored, and to identify sub-groups of patients who have higher healthcare costs. The aim of this study was to describe the healthcare costs for patients with HNC in Queensland, Australia and explore factors associated with increased costs, as well as to explore the differences in costs for regional and rural patients compared with their metropolitan counterparts.

## Methods

### Study design and setting

This study used a sub-population of patients (mucosal head and neck squamous cell carcinoma only) from the ‘Lifetime Costs of Surviving Cancer – A Queensland Study (COS-Q).’ A detailed description of the methodology has previously been described, and is summarised below [8]. The Strengthening the Reporting of Observational Studies in Epidemiology (STROBE) guidelines were followed in preparation of this manuscript [9].

Ethics approval was granted from the University of the Sunshine Coast Human Research Ethics Committee (USC HREC Approval A221792) and the Australian Institute of Health and Welfare (AIHW) Human Research Ethics Committee (EO2017/3/348). The approval for Queensland data extraction and linkage was obtained under the Public Health Act 2005 (grant RD007281). Data linkage was performed by the state (Statistical Services Brach in Queensland Health) and national departments (AIHW), before being transferred to the Secure Unified Research Environment (SURE).

### Australian healthcare setting

Australia has a universal tax funded health insurance program known as Medicare. The major components of this include coverage of healthcare provided in public hospitals and some services in private hospitals, subsidies for outpatient medical services (Medicare Benefits Schedules) and subsidies for medications (Pharmaceutical Benefits Scheme, PBS). Private health insurance can also be purchased to allow additional coverage (such as dental and allied health) or to provide coverage for admissions to private hospitals. People can face out of pocket (OOP) costs for outpatient medical services when the fee charged is higher than the Medicare benefit rebate [10].

### Study participants

As described in the COS-Q study protocol, Queensland residents diagnosed with a first primary cancer between January 1997 and December 2015 were identified from the Queensland Cancer Register (QCR). Patients who died prior to 2013 were excluded from the cohort (n = 130,102), as healthcare cost data was only available from 2013 to 2016 (discussed below). Therefore, the cohort consisted of all patients diagnosed between January 1997 and December 2015 who were alive and incurred healthcare costs in the years 2013 to 2016.

Patients with HNSCC were identified using the World Health Organisation’s International Classification of Diseases for Oncology, 3rd edition (ICD-03). The QCR also records tumour morphology, and this was used to identify patients with SCC. The ICD-03 codes encompassing tumours of the nasopharynx, oropharynx, oral cavity, hypopharynx and larynx were used (C01-6, C08-14 and C30-32) to identify the cohort of interest.

### Study variables

Healthcare costs incurred between January 1st 2013 and 31st of December 2016 were investigated. Direct costs associated with episodes of care were only available from mid-2012 and as such the period from 2013 to 2016 was the most accurate and recent costing data available from the dataset. Patients who died in one of these years were still included in the analysis, and partial costs for that year were calculated. A bottom-up costing approach was used which incorporated patient level records on hospital admissions, emergency department presentations, pharmaceutical use and medical and allied health services to generate a total cost. Costs were reported in 2016 Australian dollars (AU$). Healthcare costs were associated with all episodes of care and could not be separated into cancer-related and non-cancer related costs. The components of the cost data are summarised below and were calculated on an individual patient basis as a total cost per year:

- Emergency department (ED) costs: Data from the Emergency Department Information System (EDIS)
- Hospitalisation costs: Data from the Queensland Hospital Admitted Patient Data Collection (QHAPDC)
- Medical and allied health service costs: Data from the Medicare Benefits Schedule (MBS), which represents Medicare claims records. Benefits paid was the amount that healthcare providers received from the government. Patient OOP costs were calculated as the difference between the fee charged by a provider and the benefits paid. The total cost of medical and allied health services was calculated as the sum of the patient OOP and benefits paid.
- Pharmaceutical costs: Data from the PBS. Benefit paid was the amount that the government contributed towards the cost. The benefits paid and the patient OOP was combined to form the total cost of pharmaceuticals.

### Statistical analysis

Stata version 16 was used for analysis of the data, with the level of significance set at p < 0.05. A mean annual cost was calculated per patient for each of the four groups of costs. A total mean cost per patient per year was calculated by totalling the costs incurred by each patient and dividing by the number of years that a cost was incurred. Costs were reported as mean and standard deviation, with zero costs included. To determine how costs are affected by time since diagnosis, the mean cost was calculated for 0–1, 2–4, 5–9, 10–14 and 15–20 years post diagnosis.

Patients were classified as being in a major city or regional (inner and outer regional) or remote (remote and very remote) geographical area based on the Accessibility/Remoteness Index of Australia (ARIA) classification [11]. Mean cost per patient per year was compared between these three locations, with a Kruskal–Wallis test used to assess for differences between groups. Socio-demographic and clinical factors associated with healthcare costs were assessed using generalised linear models with a log-link function and gamma distribution. Dependent variables included age at diagnosis (continuous variable), year of diagnosis, HNC subsite, sex, country of birth, marital status, and location of residence (major city, regional or remote).

### Second primary cancers

The costs associated with a second primary cancer were explored by identifying patients who had a second diagnosis of cancer recorded in the Queensland Cancer Registry (between January 1997 and December 2015). The total cost of care for those patients (between 2013 and 2016) who were diagnosed with a second primary cancer was then calculated and compared with the group of patients who did not develop a second primary cancer during the same timeframe.

Grouping of second primary malignancies into categories (lung, HNC, other) was performed based on the most recent cancer diagnosed for those who developed multiple second primaries. Cancer recurrence is not recorded in the Queensland Cancer Registry and it likely that those patients coded with second primary malignancies of the head and neck region were true second primary malignancies.

## Results

**Fig. 1:**
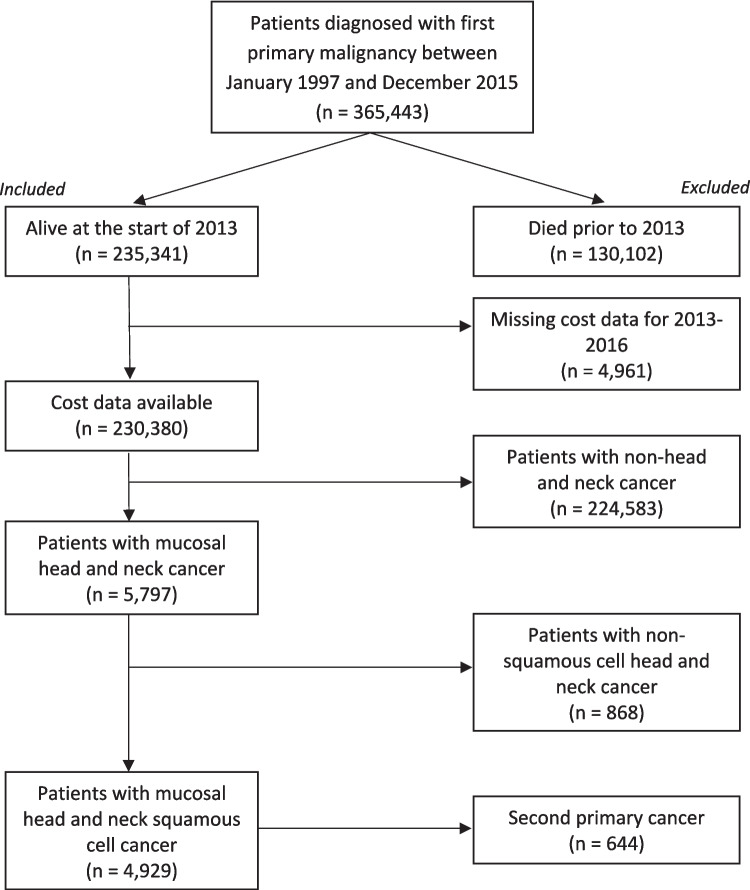
Flowchart of included patients

Using the Queensland Cancer Registry there were 365,443 patients identified who were diagnosed with a first primary malignancy between January 1997 and December 2015. A total of 230,380 patients were alive in 2013 and had available cost data. The final cohort for analysis comprised 4,929 patients with head and neck squamous cell cancer (Fig. 1).

### Demographics

Patients were predominantly male (77%), with a mean age at diagnosis of 60.3 years (SD 11.6) (Table 1). Most patients resided in a major city (54%), with 42% of patients living in regional area and 3% in a remote region. The most common HNC subsite was oropharynx (36%) followed by oral cavity (32%), and most patients were diagnosed from 2009 to 2015 (64%). For patients who died within the study period, 62% were from HNC, whilst non-cancer deaths comprised 21% and lung cancers accounted for 8%.

**Table 1:** Demographics of patient cohort

Patient demographics (*n* = 4929)	
Mean age at diagnosis (SD)*	60.3 (11.6)
*Demographics*	*N (%)*
*Sex*	
Male	3779 (77%)
Female	1069 (22%)
Unknown	81 (2%)
*Country of birth*	
Australia	3669 (74%)
Outside Australia	1260 (26%)
*Marital status*	
Married/de facto	3096 (63%)
Divorced/widowed	1256 (25%)
Never married	560 (11%)
Unknown	17 (0.34%)
*Accessibility and Remoteness Index of Australia (ARIA)*	
Major cities	2618 (53%)
Inner regional	1199 (24%)
Outer regional	890 (18%)
Remote and very remote	130 (3%)
Unknown	92 (2%)
*Socio-Economic Indexes for Areas (SEIFA)*	
1–2 (most disadvantaged)	855 (18%)
3–4	1160 (24%)
5–6	1254 (26%)
7–8	973 (20%)
9–10 (most advantaged)	584 (12%)
Unknown	22 (0.45%)
*Head and neck cancer subsite*	
Oropharynx	1783 (36%)
Oral Cavity	1553 (32%)
Nasopharynx	83 (2%)
Hypopharynx	226 (5%)
Larynx	1065 (22%)
Other	219 (4%)
*Year of diagnosis*	
1997–2002	576 (12%)
2003–2008	1217 (25%)
2009–2015	3136 (64%)
Treated with radiotherapy^#^	
Yes	1139 (67%)
No	570 (33%)
*Cause of death (n* = *743)*	
Head and neck cancer	462 (62%)
Lung cancer	61 (8%)
Other cancers	65 (9%)
Non-cancer death	155 (21%)

^*^SD standard deviation

^#^Treatment modality only able to be assessed for patients diagnosed between 2013 and 2015

### Healthcare system and patient costs

Total mean annual costs (combined cost of ED, QHAPDC, MBS and PBS) across the cohort were $21,646 (SD $31,052) per person (Table 2). Overall costs were predominantly driven by hospital admissions with a mean annual cost of $15,119 (SD $29,140) per person which was incurred by 70% of patients. More than half the cohort (58%) accessed ED during the study period, with a mean of 3.5 visits amongst those who did, at a mean cost per visit of $808 (SD $419). Similarly, 65% of patients had OOP expenses for outpatient medical or allied health services (MBS), with a mean of $571 (SD $1,312) per year. Most patients (98%) had OOP expenses for medications (PBS), with a mean of $257 (SD $249) per year.

**Table 2:** Healthcare system and patient costs

Healthcare item	Mean annual cost in $AUD (SD)
Total annual costs—ED, QHAPDC, MBS, PBS (*n* = 4929)	**$21,646 ($31,052)**
Emergency Department (*n* = 2869) Zero costs (42%)	**$917 ($1,338)**
QHAPDC—Admissions (*n* = 3433) Zero costs (30%)	**$15,119 ($29,140)**
MBS	
Benefit paid (n = 4,783) Zero costs (3%)	**$3337 ($3259)**
Out-of-pocket (*n* = 3217) Zero costs (35%)	**$571 ($1312)**
PBS (medications)	
Benefit paid (*n* = 4388) Zero costs (11%)	**$1500 ($3616)**
Out-of-pocket (*n* = 4806) Zero costs (2%)	**$257 ($249)**

Statistics reported as mean (SD), zero costs included

*ED* Emergency Department, *QHAPDC* Queensland Hospital Admitted Patient Data Collection, *MBS* Medicare Benefits Schedule, *PBS* Pharmaceutical Benefits Scheme

Overall costs were highest in the first-year post diagnosis (annual mean of $26,292 per person), compared to 2–20 years post diagnosis (Table 3). Hospital costs were substantially higher in the first-year post diagnosis ($26,371) compared to 2–20 years post diagnosis ($17,764).

**Table 3:** Overall healthcare costs stratified by time since diagnosis

Healthcare item	Mean annual cost in $AUD (SD)
**Total annual costs (ED, QHAPDC, MBS, PBS)**	
Overall annual cost (*n* = 4929)	
0–1 years (*n* = 2161)	$26,292 ($30,520)
2–4 years (*n* = 2245)	$10,990 ($25,100)
5–9 years (*n* = 1934)	$11,008 ($20,250)
10–14 years (*n* = 1083)	$11,640 ($21,348)
15–20 years (*n* = 402)	$11,977 ($19,582)
**Emergency Department**	
Overall annual cost	
0–1 years (*n* = 1165)	$1773 ($1817)
2–20 years (*n* = 1999)	$1502 ($1371)
**QHAPDC (admissions)**	
Overall annual cost	
0–1 years (*n* = 1695)	$26,371 ($36,472)
2–20 years post diagnosis (*n* = 2262)	$17,764 ($29,769)
**MBS**	
Benefit paid (*n* = 4783)	
0–1 years (*n* = 2089)	$5894 ($4223)
2–20 years (*n* = 3918)	$2320 ($2476)
Out-of-pocket (*n* = 3217)	
0–1 years (*n* = 1220)	$1267 ($2197)
2–20 years (*n* = 2605)	$735 ($1304)
**PBS (medications)**	
Benefit paid (*n* = 4388)	
0–1 years (*n* = 1914)	$1987 ($4448)
2–20 years (*n* = 2605)	$1515 ($3921)
Out-of-pocket (*n* = 4806)	
0–1 years (*n* = 2073)	$269 ($266)
2–20 years (*n* = 3881)	$253 ($251)

*ED* Emergency Department, *QHAPDC* Queensland Hospital Admitted Patient Data Collection, *MBS* Medicare Benefits Schedule, *PBS* Pharmaceutical Benefits Scheme

### Costs for patients from non-metropolitan vs metropolitan regions

Overall mean healthcare costs were higher for regional ($23,312, SD $31,897) and remote ($33,627, SD $55,264) patients compared to those who resided in a major city ($20,300, SD $28,787, p < 0.001, Table 4).

**Table 4:** Costs for patients from metropolitan vs non-metropolitan regions

Healthcare cost (SD)*	Mean annual cost stratified by location of residence			*P* value
	Major cities (*n* = 2,618)	Regional (*n* = 2,089)	Remote (*n* = 130)	
Overall costs	$20,300 ($28,787)	$23,312 ($31,897)	$33,627 ($55,264)	< 0.001
ED costs	$830 ($1229)	$1,020 ($1419)	$1,525 ($1955)	< 0.001
QHAPDC costs	$13,553 ($26,560)	$16,857 ($30,116)	$28,003 ($54,883)	< 0.001
MBS benefit	$3,450 ($3483)	$3,304 ($3004)	$2,829 ($2846)	0.056
MBS OOP	$676 ($1528)	$470 ($1013)	$194 ($708)	< 0.001
PBS benefit	$1,572 ($3521)	$1,472 ($3787)	$1,009 ($3722)	< 0.001
PBS OOP	$273 ($267)	$243 ($221)	$167 ($221)	< 0.001

*ED* Emergency Department, *QHAPDC* Queensland Hospital Admitted Patient Data Collection, *MBS* Medicare Benefits Schedule, *PBS* Pharmaceutical Benefits Scheme

^*^Reported as mean annual cost in $AUD (SD), zero costs included

There were substantial differences in QHAPDC costs between the three groups, with patients from remote ($28,003, SD $54,883) and regional ($16,857, SD $30,116) regions having higher mean annual costs per patient compared to those from major cities ($13,553, SD $26,560). Similarly, ED costs were higher in the remote ($1,525, SD $1,955) and regional ($1,020, SD $1,419) compared to those from major cities ($830, SD $1,229, p < 0.001). Mean annual OOP for both MBS and PBS were lower for patients from remote and regional locations compared to major cities (p < 0.001).

### Factors associated with increased healthcare cost

Socio-demographic factors associated with increased overall healthcare costs were found to be age at diagnosis (RR 1.02, *p* < 0.001), living in a remote (RR 1.51, *p* = 0.001) area, residing in a disadvantaged suburb (SEIFA 1–4, HR 1.16, *p* = 0.001) and being either divorced/widowed (RR 1.19, *p* < 0.001) or never married (RR 1.34, *p* < 0.001, Table 5). Participants with nasopharynx (RR 1.41, *p* = 0.027) and hypopharynx tumours (RR 1.60, *p* < 0.001) also experienced higher healthcare costs, as well as those diagnosed from 2009 to 2015 compared to those diagnosed in 1997–2002 (RR 1.26, *p* < 0.001).

**Table 5:** Factors associated with increased healthcare costs

Overall costs		
Variable	RR (95% CI)	*p* value
Age (at diagnosis)	1.02 (1.01–1.02)	< 0.001
Year of diagnosis		
1997 to 2002—reference group		
2003 to 2008	0.82 (0.71–0.95)	0.007
2009 to 2015	1.26 (1.11–1.43)	< 0.001
Tumour site		
Oral cavity—reference group		
Oropharynx	0.99 (0.90–1.09)	0.853
Nasopharynx	1.41 (1.04–1.92)	0.027
Hypopharynx	1.60 (1.32–1.94)	< 0.001
Larynx	1.07 (0.96–1.20)	0.230
Other	1.23 (1.01–1.50)	0.038
Sex		
Female—reference group		
Male	1.08 (0.98–1.19)	0.122
Location of residence (ARIA)		
Major city—reference group		
Regional	1.06 (0.97–1.15)	0.202
Remote	1.51 (1.18–1.93)	0.001
Country of birth		
Australia—reference group		
Other	1.00 (0.92–1.10)	0.956
Marital status		
Married/de facto—reference group		
Divorced/widowed	1.19 (1.09–1.30)	< 0.001
Never married	1.34 (1.18–1.53)	< 0.001
SEIFA		
5–10 (reference group)		
1–4	1.16 (1.06–1.26)	0.001

*RR* relative risk

These analyses were also repeated separately to identify associations between tested clinical and socio-demographic variables and healthcare costs for ED, QHAPDC, MBS Benefits, MBS OOP, PBS Benefits and PBS OOP (Supplementary 1). Similar socio-demographic factors (age, residing in a remote location, lower socioeconomic status, never married or divorced) were associated with both increased ED and QHAPDC costs. However, patients who were divorced or never married, as well as those from a remote location had lower costs for outpatient medical and allied health (MBS). Out-of-pocket costs for both medications (PBS) and medical/allied health services (MBS) were lower for those who were never married, from a lower socioeconomic status (SEIFA 1–4) or regional/remote location. Patients with oropharynx, nasopharynx or hypopharynx tumours had higher ED and medical/allied health (MBS) costs, whilst admission (QHAPDC) costs were lower for those with oropharyngeal tumours and higher for those with nasopharynx or hypopharynx cancers. There was no difference in OOP expenses between different tumour subsites.

### Cost of second primary cancers

Within the entire study cohort of 4929 patients, there were 644 patients who were diagnosed with a second primary malignancy (13%). Of these patients there were 129 with a second primary HNC (20%), 132 who developed lung cancer (21%) and 383 (59%) with other types of malignancies. The sub-group that developed second primary cancers experienced a substantial increase in overall healthcare costs compared to patients who did not have a second primary cancer ($46,453 vs $24,966, *p* < 0.001, Table 6). When comparing costs for those diagnosed with a second malignancy between 2013 and 2015 the costs were substantial ($99,326 vs $57,650) demonstrating the high costs associated with the treatment of multiple malignancies.

**Table 6:** Healthcare costs of second primary cancers

Variable	Total cost (SD) Second primary	Total cost (SD) Comparator group	Excess mean cost	*p* value
Second primary cancer (All)	$46,453 ($67,318) *N* = 644	$24,966 ($42,919) *N* = 4285	$21,487	< 0.001
Second primary cancer (2013–2015)	$99,326 ($70,307) *n* = 85	$57,650 ($58,263) *n* = 1624	$41,676	< 0.001

## Discussion

Costs of treatment for HNC are high, especially in the first year after diagnosis. This study identified multiple socio-demographic factors associated with increased healthcare costs (socio-economic disadvantage, living in a regional or remote location, being divorced or unmarried). Clinical factors such as having a nasopharynx or hypopharynx tumour were predictive of increased cost.

The distinct differences in healthcare costs between patients residing in non-metropolitan locations warrants further exploration. Non-metropolitan patients had substantially increased annual costs of care largely driven by increased hospital admission costs, as well as increased ED costs. Whether this was because patients from regional or rural locations presented with later stage disease requiring more intensive treatment or had other medical co-morbidities which increased hospital length of stay or number of admissions remains to be elucidated. Given cancer stage and co-morbidities were not recorded in the current data set further research is required to investigate these potential correlations. Current Australian literature evaluating differences in demographics and outcomes for patients with HNC from rural or regional locations remains heterogenous, with limited high-quality data available. A prospective study from northern Queensland suggested that patients with HNC from remote or very remote regions treated with curative intent had increased risk of disease recurrence compared to those from outer regional (35.5% vs 14.3%, p = 0.04), however there were no differences in overall survival [12]. Data comparing stage of disease presentation for patients from rural or metropolitan locations with HNC is also variable, with many registry-based studies having limited staging data available [13]. Another Australian study of 23 patients which mapped the 6-month post-acute health care needs of patients with HNC found that those from a rural location had a significantly higher number of healthcare appointments compared to those from metropolitan areas (44 vs 20, *p* = 0.012) [14]. Fragmentation of care for these patients is another issue which may lead to increased hospital admissions and cost, with a registry based Australian study finding that only 15.7% of rural patients were re-admitted to the same hospital which initially provided their care [13].

Interestingly, OOP costs for both MBS and PBS services were lower for both regional and rural patients compared to those from a major city. This could be secondary to a lower healthcare utilisation rate from reduced access to out-patient services or that in general more patients from regional/rural areas are of a lower socio-economic status and were in receipt of bulk-billed services or had reached the Medicare safety net cap after which patients are not required to contribute OOP payments. However, the fact that patients from regional/remote areas also had lower MBS benefits and a trend towards a lower PBS benefit may indicate a lower outpatient healthcare utilisation in this group, particularly in remote locations. Importantly, the OOPs reported in this study do not consider indirect costs such as travel and accommodation which has been shown to substantially increase costs for those from regional and rural Australia [15]. Our previous research demonstrated that participants living in rural locations (defined as greater than 100 km from the treating hospital) had higher out of pocket expenses within the first 12 months of HNC diagnosis (AUD $2655 vs $730, *p* = 0.001) [15]. Similar trends were observed for patients living in a lower socio-economic suburb, with higher hospitalisation and ED costs but lower OOP costs.

Whilst treatment modality or cancer staging was not available in this dataset it is possible that the increased healthcare costs for patients with hypopharyngeal cancers is secondary to the increased likelihood of advanced stage disease, requirement for multi-modality treatment or high risk of recurrent disease [16]. A study performed in the USA suggested that patients receiving multi-modal HNC treatments had approximately double the healthcare costs of those requiring single modality treatments, with the highest cost of care in the group treated with surgery, radiation and chemotherapy [5].

Patients with HNC from a lower socio-economic background may have a higher risk of co-morbidities or present with advanced disease which could contribute towards the increased healthcare costs in this cohort. Multiple studies within the literature have suggested a correlation between lower socioeconomic status and an increased likelihood of later stage HNC at diagnosis [17, 18]. The increased healthcare costs for patients who were divorced or never married could be due to a number of reasons. Other studies have suggested that patients with head and neck cancer who are not married are more likely to have worse survival compared to patients who were married, as well as being more likely to present with later stage disease [19]. Both of these factors are likely to contribute towards increased healthcare costs. Previous literature has also suggested that patients with HNC who were not married had worse financial toxicity [15, 20].

This study suggests that patients with HNC are at high risk of second primary malignancies, with 13% of the cohort developing a second cancer. These findings are consistent with the literature, with a prior meta-analysis demonstrating an incidence of second primary malignancy of 13.2% (95% CI 11.56 to 14.84) amongst patients with HNC [21]. As expected, the costs associated with treatment of patients with second primary malignancies are high, particularly in the initial treatment stage, as demonstrated by the substantially elevated costs for patients who developed a second malignancy between 2013 and 2015. Preventative measures such as smoking, and alcohol cessation should therefore be strongly encouraged within HNC survivors to reduce the development of second primary cancers.

One of the main limitations of this study was the absence of cancer staging data and medical co-morbidities of the cohort. Data was not available on treatment modalities for all patients and as such analyses comparing types of treatment was not possible. Due to limitations with the dataset, determining the costs of treatment for HNC recurrence could not be performed although it would be expected that these patients would have significantly increased costs of care. There was no data available on whether patients were treated upfront with a curative or palliative intent, and this may also influence treatment costs. Furthermore, non-Medicare subsidised ED presentations or in-patient services in private hospitals would not have been captured in this dataset, as well as OOP associated with private healthcare. As the healthcare cost data were derived from routinely collected administrative sources, it was not possible to distinguish between cancer-related and non-cancer related costs. Consequently, estimating the excess costs attributable to a cancer diagnosis could not be undertaken without a cancer-free, age-matched control group.

These findings highlight several important implications for further research and healthcare policy within the Australian healthcare setting. Costs of care are largely driven by hospitalisation costs and efforts to reduce admissions should be explored, particularly in the acute setting during and after initial cancer treatment. Whilst evidence-based strategies to reduce hospital admissions are limited in the head and neck cancer setting, potential options include prophylactic gastrostomy placement during RT when clinically indicated [22]. Strategies to reduce the risk of second malignancy such as smoking cessation, alcohol reduction and lung cancer screening may represent possible strategies to reduce these rising costs of care and should be explored. For patients who continue to smoke after their cancer diagnosis, the evidence suggests that a combination approach of pharmacological and behavioural interventions is likely to be the most effective approach [23]. Similarly, preventative efforts to reduce burden of long-term side effects secondary to RT such as osteoradionecrosis, dysphagia, carotid artery stenosis and stroke may assist in improving healthcare costs as well as improving quality of life for patients. Currently in Australia subsidised dental care is limited and is not offered to all HNC survivors. Whilst not able to be investigated as part of this study, it is likely that many patients face substantial costs for dental care or are not accessing recommended dental care due to financial concerns. The high direct costs of treatment for patients from regional and remote areas in conjunction with previously published literature showing increased indirect costs (such as travel and accommodation) are concerning [15]. Additionally, the relationship between high hospital and ED costs and low outpatient MBS and PBS utilisation for regional and rural patients should be explored to examine potential causes such as limited access to outpatient services leading to late presentations, medical co-morbidities and socio-economic disadvantage. Identifying such issues in healthcare delivery can help optimise care pathways and provide improved support for these patients. Future research assessing the financial impacts of HNC should ensure that a comprehensive assessment of both direct and indirect costs of cancer care are evaluated to ensure the overall economic burden is captured.

## Conclusion

Treatment of HNCs incurs significant direct costs to the healthcare system and patients in Australia. Patients at higher risk of increased healthcare costs include those from a regional or remote location, being never married or patients with a nasopharynx or hypopharynx primary. Second primary cancers remain prevalent amongst HNC patients and result in significant healthcare costs. Future research is needed to explore ways in which healthcare delivery can be optimised to reduce cost to both the healthcare system and patients and improve quality of care for patients with HNC.

## Data Availability

The data that support the findings of this study are available from the data custodians of each of the linked datasets. Aggregated data used for this manuscript is available from the authors upon reasonable request.
